# Re-Evaluating Neonatal-Age Models for Ungulates: Does Model Choice Affect Survival Estimates?

**DOI:** 10.1371/journal.pone.0108797

**Published:** 2014-09-29

**Authors:** Troy W. Grovenburg, Kevin L. Monteith, Christopher N. Jacques, Robert W. Klaver, Christopher S. DePerno, Todd J. Brinkman, Kyle B. Monteith, Sophie L. Gilbert, Joshua B. Smith, Vernon C. Bleich, Christopher C. Swanson, Jonathan A. Jenks

**Affiliations:** 1 Department of Natural Resource Management, South Dakota State University, Brookings, South Dakota, United States of America; 2 Wyoming Cooperative Fish and Wildlife Research Unit, Department of Zoology and Physiology, University of Wyoming, Laramie, Wyoming, United States of America; 3 Department of Biological Sciences, Western Illinois University, Macomb, Illinois, United States of America; 4 U.S. Geological Survey, Iowa Cooperative Fish and Wildlife Research Unit, Department of Natural Resource Ecology and Management, Iowa State University, Ames, Iowa, United States of America; 5 Department of Forestry and Environmental Resources, Fisheries, Wildlife, and Conservation Biology, North Carolina State University, Raleigh, North Carolina, United States of America; 6 Institute of Arctic Biology and Department of Biology and Wildlife, University of Alaska Fairbanks, Fairbanks, Alaska, United States of America; 7 Sierra Nevada Bighorn Sheep Recovery Program, California Department of Fish and Game, Bishop, California, United States of America; 8 U.S. Fish and Wildlife Service, Kulm, North Dakota, United States of America; University of Louisville, United States of America

## Abstract

New-hoof growth is regarded as the most reliable metric for predicting age of newborn ungulates, but variation in estimated age among hoof-growth equations that have been developed may affect estimates of survival in staggered-entry models. We used known-age newborns to evaluate variation in age estimates among existing hoof-growth equations and to determine the consequences of that variation on survival estimates. During 2001–2009, we captured and radiocollared 174 newborn (≤24-hrs old) ungulates: 76 white-tailed deer (*Odocoileus virginianus*) in Minnesota and South Dakota, 61 mule deer (*O. hemionus*) in California, and 37 pronghorn (*Antilocapra americana*) in South Dakota. Estimated age of known-age newborns differed among hoof-growth models and varied by >15 days for white-tailed deer, >20 days for mule deer, and >10 days for pronghorn. Accuracy (i.e., the proportion of neonates assigned to the correct age) in aging newborns using published equations ranged from 0.0% to 39.4% in white-tailed deer, 0.0% to 3.3% in mule deer, and was 0.0% for pronghorns. Results of survival modeling indicated that variability in estimates of age-at-capture affected short-term estimates of survival (i.e., 30 days) for white-tailed deer and mule deer, and survival estimates over a longer time frame (i.e., 120 days) for mule deer. Conversely, survival estimates for pronghorn were not affected by estimates of age. Our analyses indicate that modeling survival in daily intervals is too fine a temporal scale when age-at-capture is unknown given the potential inaccuracies among equations used to estimate age of neonates. Instead, weekly survival intervals are more appropriate because most models accurately predicted ages within 1 week of the known age. Variation among results of neonatal-age models on short- and long-term estimates of survival for known-age young emphasizes the importance of selecting an appropriate hoof-growth equation and appropriately defining intervals (i.e., weekly versus daily) for estimating survival.

## Introduction

Survival of young ungulates often drives annual fluctuations in population growth because adult survival is relatively constant in comparison to young [1]–[3]. Therefore, determining factors that influence the ecology and mortality of young ungulates is important for population management and understanding how pre-hunt survival affects sustainable harvest rates [4], [5]. Yet, collecting data on neonates can be challenging because of their cryptic coloration and inactivity during the first month of life; thus, making capture difficult and survival information costly to collect [4].

Regional and seasonal variation in survival rates and cause-specific mortality of young ungulates with respect to sex, age, animal density, and environmental conditions further complicates the interpretation and limits the application of results of such studies [6]–[10]. Additionally, survival and cause-specific mortality of young vary as a function of age, with marked changes occurring within the first few weeks of life [5], [11]–[14]. This strongly suggests accurate information on date of birth is critical to understanding age-dependent patterns of survival and risk to specific sources of mortality. Inaccuracies in age estimates of captured neonates could affect estimates of survival because age-at-capture determines the interval that an individual enters and exits staggered-entry models [15]–[17].

To estimate age-at-capture, researchers have developed regression models to predict age (*y*-axis) of newborn white-tailed deer (*Odocoileus virginianus*; [18]–[20]), mule deer (*O. hemionus*; [21]), and pronghorn (*Antilocapra americana*; [22]) from measurements of new-hoof growth (*x*-axis) of captive animals. The results of such models, however, may not be consistent with estimates of age from hoof-growth measurements collected from free-ranging animals [17]. Although slopes among published equations describing the relationship between age (days) as a function of the independent variable new-hoof growth (mm) often are similar, intercept terms differ, thereby producing different age estimates for the same neonate. For example, intercepts of published growth equations ranged from −8.29 to 0.66 for white-tailed neonates [17]–[20] and from −6.30 to 5.29 for mule deer neonates [17], [21].

Our first objective was to evaluate existing models for estimating age of neonates from measurements of hoof growth of known-age (≤24-hours old), wild newborns of 3 species (white-tailed deer, mule deer, and pronghorn) from study sites in California, South Dakota, and Minnesota, USA. We expected age estimates of newborns to vary among hoof-growth equations because of the variation in slopes and intercepts among models. Our second objective was to quantify the consequences of estimating age-at-capture of neonate ungulates on the resulting estimates of survival at both 30 and 120 days. We predicted that survival estimates would differ among models. Specifically, differences in estimation of age (days) at the time of capture would influence the time interval at which a neonate entered and exited the population at-risk in survival analyses.

## Materials and Methods

### Study Area

**Figure 1:**
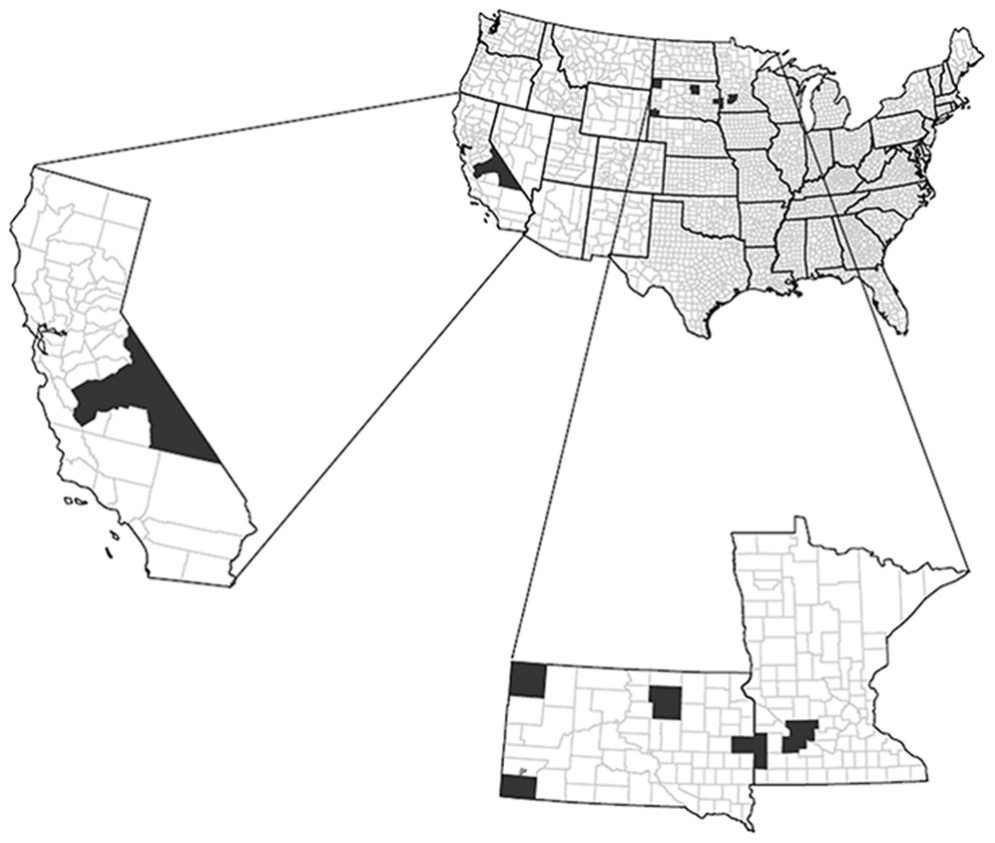
Locations of ungulate study populations in California, South Dakota, and Minnesota, USA. White-tailed deer (*Odocoileus virginianus*), mule deer (*O. hemionus*), and pronghorn (*Antilocapra americana*) study areas (shaded) in California, South Dakota, and Minnesota, USA, 2001–2009.

Our study was conducted in Minnesota (white-tailed deer), South Dakota (white-tailed deer and pronghorn), and California (mule deer), USA (Fig. 1). We captured pronghorn in western South Dakota (Fall River, Harding, and Custer counties). Wind Cave National Park (WCNP), our study area in Custer County, was located in the southern Black Hills and was enclosed by a 2.5-m woven-wire fence with cattle guards to prevent passage by ungulates [23]. Our study areas in Fall River and Harding counties were characterized by a mosaic of mixed-grass prairie interspersed with shrubs (e.g., sagebrush [*Artemisia* spp.]) and patches of forest. Topography was rolling, with occasional buttes and intermittent streams [24], [25].

We captured white-tailed deer fawns in study areas in north-central and eastern South Dakota, and in southwestern Minnesota, USA. North-central South Dakota (Edmunds and Faulk counties) was characterized by previously glaciated, rolling prairie interspersed with pothole wetland complexes, cultivated agricultural land, intermittent streams, and river floodplains [26]. The eastern South Dakota study area (Brookings County) lies within the Prairie Coteau Region formed by the Wisconsin Glaciation [27]. The Prairie Coteau historically contained numerous wetlands and in eastern South Dakota, approximately 35% were drained through anthropogenic modifications (e.g., agriculture; [25], [28]). The southwest Minnesota study area was characterized by flat-to-rolling topography [29]. Lincoln and Pipestone counties, Minnesota, are within the Prairie Coteau physiographic region, whereas Redwood and Renville counties occur within the Minnesota River Valley physiographic region. The Minnesota River Valley was a linear corridor that was heavily forested with small interspersed grassland remnants and adjacent lands comprised primarily of cultivated crops [13], [30]. Habitat in the region was fragmented and dominated by intense row-crop agriculture [31].

In California, our study area was located in the Sierra Nevada (a high-elevation mountain range) and focused on a migratory mule deer population that wintered on the western edge of the Great Basin that included Fresno, Inyo, Madera, and Mono counties. Summer range for mule deer in the Sierra Nevada occurred on both sides of the Sierra crest at elevations ranging from 2,200 to>3,000 m [9], [32]. The western slope of the summer range was substantially more mesic and dominated by the upper montane and mixed conifer vegetation zones, whereas the xeric, eastern slope of the Sierra Nevada (<2,100 m) was dominated by the sagebrush vegetation zone [9], [32].

### Adult Capture

We captured adult female white-tailed deer and mule deer on winter range using a hand-held net gun fired from a helicopter [33]. In Minnesota, female white-tailed deer were immobilized with ketamine hydrochloride (5 mg/kg IM; Ketaset; Fort Dodge Laboratories, Fort Dodge, IA, USA) and xylazine hydrochloride (1 mg/kg IM; Xylaject, Phoenix Pharmaceutical Inc., St. Joseph, MO, USA). We used yohimbine hydrochloride (0.125 mg/kg IV; Yobine, Ben Venue Laboratories, Inc., Bedford, OH, USA) as an antagonist [34]. We did not use chemical immobilization during adult mule deer capture in California [32]. White-tailed deer and mule deer females were blindfolded and hobbled prior to transport via helicopter to a central processing station.

We assumed all captured adult white-tailed deer in Minnesota were pregnant, given the high pregnancy rates for adult deer inhabiting these agricultural regions [35], [36]. We assumed that not all captured mule deer at the California site were pregnant, and determined pregnancy status and litter size of mule deer with trans-abdominal ultrasonography [37]. We captured 14 adult female white-tailed deer in Minnesota and 263 adult female mule deer in California. All white-tailed deer and 178 mule deer were fitted with vaginal implant transmitters (VIT; Model M3930, Advanced Telemetry Systems, Isanti, MN, USA) using standard procedures [30], [38], [39].

### Neonate Capture

To spatially focus our search efforts for newborns, we used VITs, pre- and post-partum behavior of radiocollared dams and post-partum behavior of unmarked dams to locate birth sites. We captured neonatal white-tailed deer during late May and early June 2001–2004 in eastern South Dakota and southwestern Minnesota [13], [30], [31], and 2007–2009 in north-central South Dakota [40]–[42]. We captured pronghorn neonates during late May and early June 2002–2005 in western South Dakota [43], and neonatal mule deer in California during mid–June through mid–July 2006–2008 [14], [44].

We monitored VITs 1–3 times daily using a very high frequency (VHF) receiver and a truck-mounted, null-peak antenna system with an electronic digital compass (C100 Compass Engine; KVH Industries, Inc., Middletown, RI, USA; [45]), Cessna 182 and 185 aircraft (Cessna Aircraft Co., Wichita, KS, USA) fitted with 2, 2-element Yagi antennas or a hand-held Yagi antenna (Advanced Telemetry Systems). When a VIT indicated a birth had occurred, we immediately (<4 hours in Minnesota, <12 hours in California) located the unit and radiocollared female. We used the location of the VIT and radiocollared female to focus a search area, and implemented intensive ground searches to locate neonates. Because we checked all VIT signals a minimum of once per day during the fawning period, time of birth was known for newborns captured ≤12 hours after receiving a signal the VIT had been expelled. Because VITs may be expelled pre-partum [39], [46], we used other indicators of age for newborns captured >12 hours after the VIT was shed and for newborns captured from dams without VITs. We classified neonates as ≤24-hrs old if they met all of the following criteria: 1) tips of hooves were soft and supple, often white, and a soft semi-gelatinous sulphur-yellow pad was still present on the bottoms of hooves and tips of dewclaws [18], 2) umbilicus was still wet and fresh in appearance [18], and 3) alarm bradycardia (includes a motionless behavioral response and decreased heart rate) and a high level of passiveness were displayed [20], [47]. We removed all neonates >24-hrs old from our analyses because we were interested in evaluating the performance of new-hoof growth equations based on known-age newborns.

We did not use VITs for white-tailed deer in eastern and north-central South Dakota, or for pronghorn in western South Dakota. Instead, we relied on post-partum behavior of females [13], [35], [42], [48]–[50] or direct observations of neonates [51] to facilitate capture. Nevertheless, we used the same 3 criteria used for females with VITs when ascertaining if neonates were ≤24-hrs-old.

We manually restrained neonates of all 3 species and carefully measured new-hoof growth (the distance from the hairline to the growth-ring line on the outer edge of the hoof [20]) to the nearest 0.1 mm using a dial caliper, and used average new-hoof growth of the 2 front hooves in subsequent analyses. The same researcher measured or trained technicians to measure new-hoof growth at each site throughout the study period. We fitted each neonate with an expandable radiocollar (model M4210, Advanced Telemetry Solutions, Isanti, MN, USA; model CB-6, Telonics Inc., Mesa, AZ, USA; model TS-37, Telemetry Solutions, Concord, CA, USA) to monitor survival. We relocated neonates daily for the first 30 days of life and then ≥1 time per week through at least 120 days old using a truck mounted null-peak antenna, Cessna 182 and 185 aircraft, or a hand-held Yagi antenna. When we detected a mortality signal, ground personnel located the collar within 12 hours (range 0.5–12 hours). We examined physical evidence at the collar-recovery site to ascertain whether the device was shed prematurely or the neonate died. We used evidence of struggle, blood (on ground or collar), or remains as indications of cause of neonate mortality. Collars attached to fencing, trees, or shrubs, and collars with all folds expanded with no evidence of mortality in the immediate area (50-m radius) were considered prematurely shed [44]. We estimated date of collar loss as the mid-point between date last known alive and date of mortality signal.

Animal handling methods used in this project followed guidelines recommended by the American Society of Mammalogists [52] and were approved by the Institutional Animal Care and Use Committee at South Dakota State University (Approval nos. 04-A009, 00-A038, 02-A037, 02-A043, 02-A001, 02-A002) and an Independent Institutional Animal Care and Use Committee at Idaho State University (Protocol number 650-0410). Data collection was authorized by California Department of Fish and Wildlife, South Dakota Department of Game, Fish and Parks, and Minnesota Department of Natural Resources. All data were collected on private land with permission of individual landowners as well as public land with permission of California Department of Fish and Wildlife, South Dakota Department of Game, Fish and Parks, Minnesota Department of Natural Resources, and the U.S. Fish and Wildlife Service. Field studies did not involve endangered or protected species.

### Statistical analysis

For white-tailed deer, we evaluated hoof-growth equations of Haugen and Speake (hereafter Haugen and Speake; [18]), Sams et al. (hereafter Sams; [19]), Brinkman et al. (hereafter Brinkman; [20]), and Haskell et al. (hereafter Haskell; [17]). For mule deer, we evaluated the equations of Robinette et al. (hereafter Robinette; [21]) and Haskell et al. (hereafter Haskell; [17]). For pronghorn, we evaluated the Tucker and Garner (hereafter Tucker and Garner; [22]) equation (Table 1). We evaluated hoof-growth equations by: 1) comparing and contrasting age of known-age (≤24-hours old) newborns with age estimates using each hoof-growth equation based on new-hoof growth measurements, and 2) comparing age-specific estimates of 30-day and 120-day survival from known ages against age estimated from each hoof-growth equation. We analyzed each species separately and used analysis of variance (ANOVA) and Tukey-Kramer pairwise comparisons to determine differences in estimates of new-hoof growth among study sites and years, and controlled for study site by blocking to account for possible measurement bias by investigators. We conducted statistical tests using SAS version 9.3 [53], with an experiment-wide error rate of 0.05.

**Table 1:** Intercept and slope for published neonatal-age models used to estimate age (days) of neonatal white-tailed deer, mule deer, and pronghorn based on measurements of new hoof growth (mm).

Species	Model^a^	Intercept	Slope
White-tailed deer	Haugen and Speake	0.66	2.20
	Sams	−8.29	3.66
	Brinkman	−5.73	3.14
	Haskell	0.57	3.87
Mule deer	Robinette	−6.30	2.55
	Haskell	5.29	2.54
Pronghorn	Tucker and Garner	0.89	2.34

a Models are those of Brinkman [20], Haskell [17], Sams [19], Haugen and Speake [18], Robinette [21], or Tucker and Garner [22].

We evaluated the effect of age estimates from the various hoof-growth equations proposed for each species on survival estimates using known-fate models in Program MARK [54] with the logit-link function to model survival of neonates to 30 and 120 days-of-age. The focus of known-fate models is the probability of surviving an interval between sampling occasions. The sampling or detection probabilities are assumed to be 1 because the animal is radio-tagged [55]. The model is a product of simple binomial likelihoods and the precision of known-fate models is typically high [55]. We selected 30-day survival because research on neonates <1 month of age provides critical information regarding reproduction, sex ratios, mortality, movements, and behavior [48]. We selected 120-day survival to approximate summer survival of young ungulates. For the known-aged model, all neonates entered the analysis during the first interval because only neonates known to be <24 hrs old were included in our analysis. In contrast, when estimating age using hoof-growth equations, newborns that were estimated to be 0–24 hrs old at capture entered the survival analysis during the first interval, and newborns estimated to be 24–48 hrs old at capture entered the analysis in the second interval, and so on. When using hoof-growth equations with negative intercepts [19]–[21], we truncated estimated age of neonates to zero when estimates were <0-days old. We right-censored all animals that prematurely shed collars because censoring likely was independent of the fate of the animal [44]. During daily monitoring, we right-censored animals on the day the collar was retrieved. During less intense monitoring (i.e., ≥1 time per week), we right-censored animals at the mid-point between last relocation and day the collar was retrieved.

We evaluated survival models that assumed daily survival was constant throughout the 30-day or 120-day period, and models that allowed daily survival to vary randomly among days (daily survival) or among weeks (weekly survival; [54], [55]). For each newborn, we created an encounter history using the neonate's actual age as well as an encounter history for the neonate's age estimated using each hoof-growth equation. We evaluated potential differences in survival as a function of estimated age by comparing models with different group assignments. For example, for mule deer, we contrasted models that considered survival varied among groups (known-age [KA], Robinette [R], and Haskell [H] models; KA, R, H), with a model that considered survival of known-age neonates different from those estimated by hoof-growth equations (assumed survival differed between the 2 groups with survival equal between Robinette and Haskell equations; KA, R = H), with a model that considered survival was constant (assumes survival was similar between the groups; KA = R = H), and so forth. For mule deer and pronghorn, we modeled all combinations of group assignments for known age newborns with ages estimated from hoof-growth equations. Because there were 4 hoof-growth equations for white-tailed deer, we selected model combinations based on similarities in mean age estimates calculated from the models. For instance, model KA,B,S,HS,H tested the hypothesis that survival differed among the 5 groups (known-age, Brinkman [B], Sams [S], Haugen and Speake [HS], and Haskell [H]), whereas KA = B = S, HS = H assumed survival to be the same for known-age neonates and those aged using the Brinkman and Sams equations but differed from those aged with the Haugen and Speake and Haskell equations (which were similar). We used Akaike's Information Criterion corrected for small sample size (AIC*_c_*) to select models that best described the data. We used model weights (*w_i_*) as an indication of relative support for each model, and considered models with ΔAIC*_c_* values <2 from that of the top-ranked models to be viable alternatives [56]. We used Program CONTRAST to test for differences in survival among cohorts in our top-ranked models [57].

## Results

During May–July 2001–2009, we captured 407 neonates. Of these, 76 of 204 white-tailed deer, 61 of 119 mule deer, and 37 of 142 pronghorn were estimated at ≤24-hrs old and thus, were used in subsequent analyses. We captured 18 white-tailed deer and all 61 mule deer with the aid of VITs. All others were captured via postpartum behavior of females or direct observation of the birth of neonates. New-hoof growth was similar (*t_34_* = −1.44, *P* = 0.26) between white-tailed deer neonates located using VITs and those located using more traditional methods. Estimated age of newborns generated from existing hoof-growth equations varied by >15 days for white-tailed deer, >20 days for mule deer, and >10 days for pronghorn (Table 2). Based on known-age of newborns, accuracy (i.e., the proportion of neonates assigned to the correct age) was greatest for the Brinkman and Sams equations for aging neonatal white-tailed deer (Table 3). For mule deer, the Robinette model was more accurate than the Haskell model (Table 3). Accuracy of the Tucker and Garner model was 0.0% for known-age newborn pronghorns (Table 3).

**Table 2:** Mean, standard error (SE), and range of age estimates (days) of newborn white-tailed deer (*n* = 71), mule deer (*n* = 61), and pronghorn (*n* = 37) in Minnesota, South Dakota, and California, USA, 2001–2009.

Species	Model^a^	Mean	SE	Range
White-tailed deer^b^	Brinkman	1.0	0.2	−1.33–6.52
	Haskell	10.7	0.1	8.85–15.20
	Sams	−0.4	0.2	−3.16–6.00
	Haugen and Speake	5.4	0.1	3.74–9.24
Mule deer^c^	Haskell	15.1	0.2	12.39–19.81
	Robinette	3.5	0.2	0.82–8.27
Pronghorn^d^	Tucker and Garner	6.4	0.2	3.10–10.2

Age estimates were generated from published neonatal-age models.

a Models were obtained from Brinkman [20], Haskell [17], Sams [19], Haugen and Speake [18], Robinette [21], or Tucker and Garner [22].

b Means differed (*P*<0.05) among all model results and from known-age young.

c Means differed (*P*<0.05) between model results and known-age young.

d Means differed (*P*<0.05) between model results and known-age young.

**Table 3:** Estimated accuracy (%) of neonatal age-classification equations for newborn white-tailed deer (*n* = 71), mule deer (*n* = 61), and pronghorn (*n* = 37) in Minnesota, South Dakota, and California, USA, 2001–2009.

Species	Model^a^	Age-estimate accuracy^b^ (%)			
		0 days	1 day	2 days	3 days
White-tailed deer	Brinkman	39.4	80.3	90.1	91.5
	Haskell	0.0	0.0	0.0	0.0
	Sams	9.9	66.2	88.7	97.2
	Haugen and Speake	0.0	0.0	0.0	0.0
Mule deer	Haskell	0.0	0.0	0.0	0.0
	Robinette	3.3	4.9	18.0	54.1
Pronghorn	Tucker and Garner	0.0	0.0	0.0	2.7

Age estimates were generated from published neonatal-age models.

a Models were obtained from Brinkman [20], Haskell [17], Sams [19], Haugen and Speake [18], Robinette [21], or Tucker and Garner [22].

b Accuracy was defined as proportion of neonates assigned to the correct age and classified within 1, 2, and 3 days of their true age.

Mean new-hoof growth of neonatal white-tailed deer differed among study sites (*F_3_,_67_* = 19.47, *P*<0.001). Neonates had larger new-hoof growth in Redwood and Renville counties (2.70 mm, 95% CI = 2.43–2.97, *n* = 17) than at other sites (Lincoln and Pipestone counties: 1.98 mm, 95% CI = 1.61–2.35, *n* = 6; Brookings County: 1.92 mm, 95% CI = 1.78–2.06, *n* = 12; Edmunds and Faulk counties: 1.98 mm, 95% CI = 1.92–2.04, *n* = 36). Mean new-hoof growth of neonatal pronghorn was similar among study sites (*F_2,34_* = 2.09, *P* = 0.139). Mean new-hoof growth of neonatal white-tailed deer was similar (*F_6,64_* = 1.77, *P* = 0.12) among years. However, mean new-hoof growth of neonatal mule deer differed (*F_2,58_* = 3.54, *P* = 0.04) among all three years; mean new-hoof growth was 3.61 mm (95% CI = 3.41–3.81, *n* = 19), 3.81 mm (95% CI = 3.65–3.97, *n* = 18), and 4.05 mm (95% CI = 3.78–4.32, *n* = 24) in 2006, 2007, and 2008, respectively. Mean new-hoof growth measurements of neonatal pronghorn differed (*F_3,33_* = 5.08, *P* = 0.005) among years (2002: 2.32 mm, 95% CI = 2.16–2.48, *n* = 21; 2003: 2.39 mm, 95% CI = 2.04–2.74, *n* = 12; 2004: 3.99 mm, *n* = 1; 2005: 1.86 mm, 95% CI = 1.39–2.33, *n* = 3), but likely was influenced by limited sample size during 2004 and 2005.

**Figure 2:**
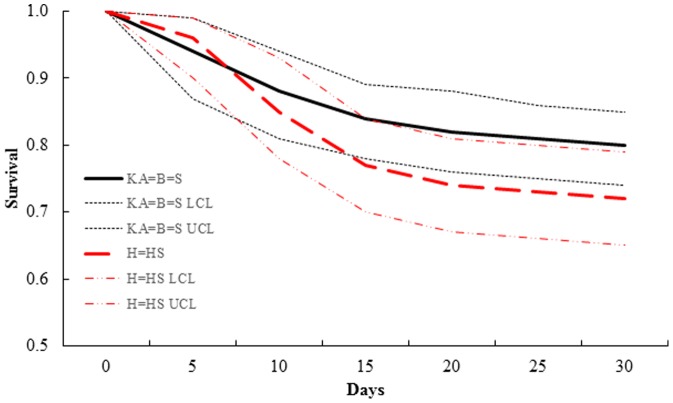
Candidate models of 30-day survival of white-tailed deer (*Odocoileus virginianus*) neonates in South Dakota and Minnesota, USA. Survival estimates up to 30 days from top-ranked model (KA = B = S, H = HS) of white-tailed deer (*Odocoileus virginianus*) neonates (*n* = 71), South Dakota and Minnesota, USA, 2001–2009. The top-ranked model indicated similar survival among known-age (KA) neonates and those aged using the Brinkman (B) and Sams (S) hoof-growth equations, which differed from neonates aged using the Haskell (H) and Haugen and Speake (HS) hoof-growth equations; survival was similar between Haskell and Haugen and Speake equations. Equations were obtained from Brinkman [20], Sams [19], Haskell [17] and Haugen and Speake [18].

The top-ranked 30-day survival model (KA = B = S, H = HS_weekly survival; *w_i_* = 0.96) for white-tailed deer indicated there were differences in survival estimates among hoof-growth equations (Table 4). The model indicated survival for known-age neonates and those with age estimated using Brinkman or Sams equations were similar (0.80, SE = 0.03, 95% CI = 0.74–0.85), but differed marginally (χ^2^_1_ = 2.56, *P* = 0.11) from those with age estimated using Haskell and Haugen and Speake (0.72, SE = 0.04, 95% CI = 0.65–0.79) equations (Fig. 2). All remaining 30-day survival models for white-tailed deer were >2 ΔAIC*_c_* from the top-ranked model. Conversely, the top-ranked survival model for 120 days post-birth (KA = B = S = H = HS_weekly survival; *w_i_* = 0.99) indicated similar survival among hoof-growth equations and known ages of fawns (Table 5). All remaining white-tailed deer survival models up to 120-days old were >2 ΔAIC*_c_* from the selected model.

**Table 4:** Survival models using Program MARK for white-tailed deer from birth to 30 days using known-age newborn fawns and ages estimated from published neonatal-age models based on new-hoof growth, South Dakota and Minnesota, USA, 2001–2009.

Model^a^	Temporal scale^b^	AIC*_c_* c	ΔAIC*_c_* d	*w_i_* e	*K* f	Deviance
{KA = B = S, H = HS}	Weekly	668.21	0.00	0.96	8	138.73
{KA = B = S, H = HS}	Constant	674.49	6.29	0.04	2	175.03
{KA = B = H = S = HS}	Daily	692.16	23.95	0.00	30	88.47
{KA = B = H = S = HS}	Weekly	692.73	24.52	0.00	4	141.26
{KA, B = H = S = HS}	Weekly	699.86	31.65	0.00	8	140.38
{KA, B, H, S, HS}	Weekly	706.80	38.59	0.00	20	123.24
{KA, B = H = S = HS}	Daily	711.21	43.00	0.00	60	46.85
{KA = B = H = S = HS}	Constant	722.50	54.29	0.00	1	177.03
{KA, B = H = S = HS}	Constant	724.46	56.26	0.00	2	177.00
{KA = B = S, H = HS}	Daily	727.64	59.44	0.00	60	63.29
{KA, B, H, S, HS}	Constant	730.33	62.12	0.00	5	176.85
{KA, B, H, S, HS}	Daily	849.04	180.84	0.00	150	0.00

Models compared and contrasted survival rates among groups and time-dependency.

a KA = known-age newborn fawns, B = Brinkman [20], H = Haskell [17], S = Sams [19], or HS = Haugen and Speake [18] neonatal-age model.

b Temporal scale represents constant, daily, or weekly survival among intervals.

c Akaike's Information Criterion corrected for small sample size [55].

d Difference in the AIC*_c_* value of the top-ranked model and that of the model under consideration.

e Akaike weight [55].

f Number of parameters.

**Table 5:** Survival models using Program MARK for white-tailed deer from birth to 120 days using known-age newborn fawns and ages estimated from published neonatal-age models based on new-hoof growth, South Dakota and Minnesota, USA, 2001–2009.

Model^a^	Temporal scale^b^	AIC*_c_* c	ΔAIC*_c_* d	*w_i_* e	*K* f	Deviance
{KA = B = S = H = HS}	Weekly	1,449.79	0.00	0.99	16	352.05
{KA, B = H = S = HS}	Weekly	1,459.88	10.09	0.01	32	330.09
{KA = B = S, H = HS}	Weekly	1,472.59	22.80	0.00	32	342.80
{KA = B = S = H = HS}	Constant	1,515.32	65.53	0.00	1	447.59
{KA = B = S = H = HS}	Daily	1,517.06	67.27	0.00	120	210.43
{KA, B = S = H = HS}	Constant	1,517.30	67.52	0.00	2	447.58
{KA = B = S, H = HS}	Constant	1,517.32	67.53	0.00	2	447.59
{KA, B, S, H, HS}	Constant	1,523.29	73.50	0.00	5	447.56
{K, B, H, S, HS}	Weekly	1,580.53	130.74	0.00	80	354.40
{KA, B = H = S = HS}	Daily	1,669.56	219.77	0.00	240	120.20
{KA = B = S, H = HS}	Daily	1,691.78	241.99	0.00	240	142.42
{KA, B, H, S, HS}	Daily	2,288.65	838.86	0.00	600	0.00

Models compared and contrasted survival rates among groups and time-dependency.

a KA = known-age newborn fawns, B = Brinkman [20], H = Haskell [17], S = Sams [19], or HS = Haugen and Speake [18] neonatal-age model.

b Temporal scale represents constant, daily, or weekly survival among intervals.

c Akaike's Information Criterion corrected for small sample size [55].

d Difference in the AIC*_c_* value of the top-ranked model and that of the model under consideration.

e Akaike weight [55].

f Number of parameters.

**Figure 3:**
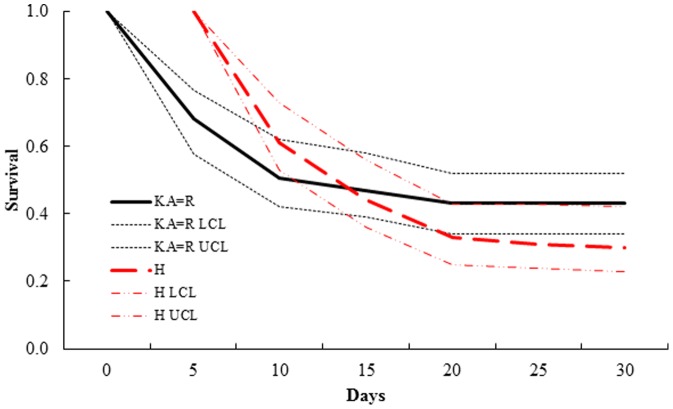
Candidate models of 30-day survival of mule deer (*Odocoileus hemionus*) neonates in California, USA. Survival estimates up to 30 days from top-ranked model (KA = R, H) of mule deer (*Odocoileus hemionus*) neonates (*n* = 61), California, USA, 2005–2007. The top-ranked model indicated similar survival between known-age (KA) neonates and those aged using the Robinette (R) hoof-growth equation, which differed from neonates aged using the Haskell (H) hoof-growth equation. Equations were obtained from Haskell [17] and Robinette [21].

**Figure 4:**
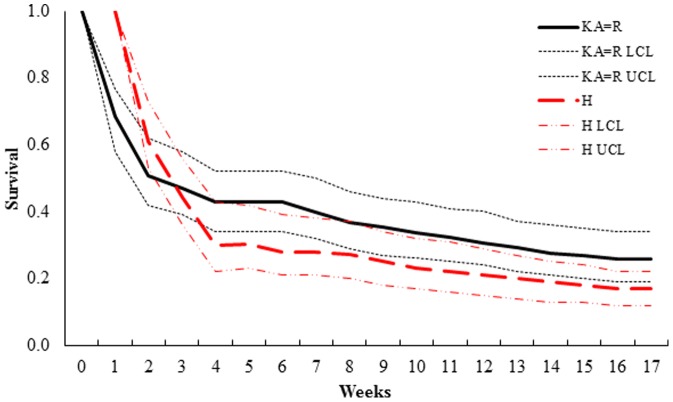
Candidate models of 120-day survival of for mule deer (*Odocoileus hemionus*) neonates in California, USA. Survival estimates up to 120 days from top-ranked model (KA = R, H) of mule deer (*Odocoileus hemionus*) neonates (*n* = 61), California, USA, 2005–2007. The top-ranked model indicated similar survival between known-age (KA) neonates and those aged using the Robinette (R) hoof-growth equation, which differed from neonates aged using the Haskell (H) hoof-growth equation. Equations were obtained from Haskell [17] and Robinette [21].

For mule deer, the top-ranked 30-day survival model (KA = R, H_weekly survival; *w_i_* = 0.93) indicated that survival estimates were similar between known-age newborns and neonates aged using the Robinette equation, but they differed marginally (χ^2^_1_ = 3.38, *P* = 0.07) from those generated from the Haskell equation (Table 6). Estimated 30-day survival for known-age newborns and those derived from the Robinette equation was 0.43 (SE = 0.05, 95% CI = 0.34–0.52), whereas estimated survival using the Haskell equation was 0.30 (SE = 0.05, 95% CI = 0.21–0.39; Fig. 3). All remaining 30-day survival models for mule deer were>2 ΔAICc from the top-ranked model. The top-ranked 120-day survival model (KA = R, H_weekly survival; *w_i_* = 0.56) indicated differences in survival estimates among hoof-growth equations and known-age newborns (Table 7). The model indicated survival estimates for known-age neonates and those with age estimated with the Robinette equation were the same (0.26, SE = 0.04, 95% CI = 0.19–0.34), but differed marginally (χ^2^_1_ = 3.24, *P* = 0.07) from those derived using the Haskell model (0.17, SE = 0.03, 95% CI = 0.12–0.22; Fig. 4). Also, we considered a competing model (KA = H = R_weekly survival; *w_i_* = 0.44), which indicated no difference in survival estimates among hoof-growth equations and known-age neonates. However, 95% confidence intervals of β-estimates overlapped zero; therefore, we eliminated this model from consideration [12], [13].

**Table 6:** Survival models using Program MARK for fawn mule deer from birth to 30 days using known-age newborn fawns and ages estimated from published neonatal-age models based on new-hoof growth, California, USA, 2005–2007.

Model^a^	Temporal scale^b^	AIC*_c_* c	ΔAIC*_c_* d	*w_i_* e	*K* f	Deviance
{KA = R, H}	Weekly	762.71	0.00	0.93	8	68.24
{KA, H, R}	Weekly	769.91	7.20	0.03	12	67.38
{KA = H = R}	Weekly	770.06	7.35	0.02	4	83.63
{KA, H = R}	Weekly	770.42	7.70	0.02	8	75.94
{KA = H, R}	Weekly	774.17	11.46	0.00	8	79.70
{KA = H = R}	Constant	785.97	23.26	0.00	1	105.55
{KA = R, H}	Constant	786.18	23.47	0.00	2	103.75
{KA, H = R}	Constant	787.29	24.58	0.00	2	104.87
{KA = H, R}	Constant	787.82	25.11	0.00	2	105.39
{KA, H, R}	Constant	788.13	25.42	0.00	3	103.70
{KA = H = R}	Daily	809.24	46.52	0.00	30	70.12
{KA, H = R}	Daily	826.13	63.42	0.00	60	24.96
{KA = H, R}	Daily	828.89	66.18	0.00	60	27.72
{KA = R, H}	Daily	840.36	77.65	0.00	60	39.19
{KA, H, R}	Daily	864.65	101.93	0.00	90	0.00

Models compared and contrasted survival rates among groups and time-dependency.

a KA = known-age newborn, R = Robinette [21], and H = Haskell [17] neonatal-age model.

b Temporal scale represents constant, daily, or weekly survival among intervals.

c Akaike's Information Criterion corrected for small sample size [55].

d Difference in the AIC*_c_* value of the top-ranked model and that of the model under consideration.

e Akaike weight [55].

f Number of parameters.

**Table 7:** Survival models using Program MARK for fawn mule deer from birth to 120 days using known-age newborn fawns and ages estimated from published neonatal-age models based on new-hoof growth, California, USA, 2005–2007.

Model^a^	Temporal scale^b^	AIC*_c_* c	ΔAIC*_c_* d	*w_i_* e	*K* f	Deviance
{KA = R, H}	Weekly	1240.64	0.00	0.56	32	200.95
{KA = H = R}	Weekly	1241.11	0.48	0.44	16	233.61
{KA, H = R}	Weekly	1254.22	13.58	0.00	32	214.53
{KA = H, R}	Weekly	1261.78	21.15	0.00	32	222.09
{KA, H, R}	Weekly	1284.33	43.69	0.00	48	212.34
{KA = R = H}	Constant	1318.66	78.03	0.00	1	341.22
{KA = R, H}	Constant	1320.53	79.89	0.00	2	341.08
{KA, H = R}	Constant	1320.61	79.98	0.00	2	341.17
{KA = H, R}	Constant	1320.64	80.01	0.00	2	341.20
{KA, H, R}	Constant	1322.53	81.89	0.00	3	341.08
{KA = H = R}	Daily	1357.58	116.94	0.00	120	138.74
{KA, H = R}	Daily	1524.23	283.60	0.00	240	55.08
{KA = H, R}	Daily	1525.95	285.31	0.00	240	56.79
{KA = R, H}	Daily	1538.79	298.15	0.00	240	69.64
{KA, H, R}	Daily	1726.70	486.06	0.00	360	0.00

Models compared and contrasted survival rates among groups and time-dependency.

a KA = known-age newborn, R = Robinette [21], and H = Haskell [17] neonatal-age model.

b Temporal scale represents constant, daily, or weekly survival among intervals.

c Akaike's Information Criterion corrected for small sample size [55].

d Difference in the AIC*_c_* value of the top-ranked model and that of the model under consideration.

e Akaike weight [55].

f Number of parameters.

The top-ranked 30-day (KA = TG; *w_i_* = 0.69) and 120-day (KA = TG_weekly survival; *w_i_* = 1.00) models for pronghorn indicated no difference in survival between known-age newborns and those with ages derived from the Tucker and Garner equation (Table 8, 9). All other pronghorn survival models were >2 ΔAIC*_c_* from the top-ranked 30- and 120-day models.

**Table 8:** Survival models using Program MARK for pronghorn from birth to 30 days using known-age newborn fawns and ages estimated from published neonatal-age models based on new-hoof growth, western South Dakota, USA, 2002–2005.

Model^a^	Temporal scale^b^	AIC*_c_* c	ΔAIC*_c_* d	*w_i_* e	*K* f	Deviance
{KA = TG}	Constant	295.71	0.00	0.69	1	54.11
{KA, TG}	Constant	297.72	2.01	0.25	2	54.11
{KA = TG}	Weekly	301.05	5.34	0.05	4	53.42
{KA, TG}	Weekly	306.36	10.64	0.00	8	50.65
{KA = TG}	Daily	317.86	22.15	0.00	30	16.98
{KA, TG}	Daily	364.73	69.01	0.00	60	0.00

Models compared and contrasted survival rates among groups and time-dependency.

a KA = known-age newborn and TG = Tucker and Garner [22] neonatal-age model.

b Temporal scale represents constant, daily, or weekly survival among intervals.

c Akaike's Information Criterion corrected for small sample size [55].

d Difference in the AIC*_c_* value of the top-ranked model and that of the model under consideration.

e Akaike weight [55].

f Number of parameters.

**Table 9:** Survival models using Program MARK for pronghorn from birth to 120 days using known-age newborn fawns and ages estimated from published neonatal-age models based on new-hoof growth, western South Dakota, USA, 2002–2005.

Model^a^	Temporal scale^b^	AIC*_c_* c	ΔAIC*_c_* d	*w_i_* e	*K* f	Deviance
{KA = TG}	Weekly	391.94	0.00	1.00	16	76.37
{KA, TG}	Weekly	420.49	28.55	0.00	32	72.57
{KA = TG}	Constant	422.42	30.48	0.00	1	136.97
{KA, TG}	Constant	424.41	32.47	0.00	2	136.96
{KA = TG}	Daily	555.18	163.24	0.00	120	25.20
{KA, TG}	Daily	790.19	398.25	0.00	240	0.00

Models compared and contrasted survival rates among groups and time-dependency.

a KA = known-age newborn and TG = Tucker and Garner [22] neonatal-age model.

b Temporal scale represents constant, daily, or weekly survival among intervals.

c Akaike's Information Criterion corrected for small sample size [55].

d Difference in the AIC*_c_* value of the top-ranked model and that of the model under consideration.

e Akaike weight [55].

f Number of parameters.

## Discussion

Variability in estimates of birth date and neonatal age affected estimates of survival generated from staggered-entry models [16], [17]. This was especially true when high rates of perinatal mortality occurred because estimates of age-at-capture determine the intervals at which neonates entered and exited survival analyses. Our results indicated that variability in estimates of age-at-capture based on neonatal-age models that were applied to known-age newborns (≤24-hours old) affected estimates of 30-day survival for both white-tailed deer and mule deer, and estimates of 120-day survival for the latter species. Model results indicated no variability for short- or long-term survival estimates for pronghorn. However, the relatively low ΔAIC*_c_* value of the second-ranked 30-day survival model suggests a similar influence on this species. Age estimates for newborns varied by >10 days for white-tailed deer and >15 days for mule deer compared with >6 days for pronghorn. The marked differences in age estimates for neonates among neonatal-age models when combined with high rates of early mortality likely led to the significant differences in 30-day survival estimates for both deer species and 120-day survival estimates for mule deer. Over longer time periods, survival estimates of white-tailed deer and pronghorn were not influenced greatly by methods of age estimation. Some hoof-growth equations used to estimate age of neonate ungulates were imprecise, which suggests the need for caution in modeling survival at too fine a temporal scale, especially if true birth dates are unknown. Most hoof-growth equations predicted ages within 1 week of the known age of newborns. Hence, potential inaccuracies in age estimates of newborns of up to 20 days may contribute to negative bias in survival estimates and decreased fit of survival models based on a daily encounter history. Although improvements in technology and performance of VITs facilitate the capture of known-age neonates and provide the opportunity to evaluate temporal patterns in ecological phenomena, our results indicate that lack of precision in neonatal-age models should be taken into consideration when birthdate is unknown.

Variability in estimates of survival among neonatal-age models for white-tailed deer and mule deer was related to intercepts of those models (Table 1). Models with negative intercepts (i.e., Brinkman, Sams, and Robinette) and expected positive values of new-hoof growth for newborns at 0 days-of-age yielded estimates of survival up to 30 and 120 days-of-age that were similar to the known-age neonate group. Conversely, survival was lower for neonates aged using models (i.e., Haskell, Haugen and Speake) with positive intercepts. The tendency for neonatal-age models with negative intercepts to predict survival estimates similar to known-age neonates may have been influenced by the truncation of negative age estimates to that of a newborn (i.e., <24 hours-of-age at birth). Additionally, these models had lower upper ranges of age estimates (e.g., 6.0–6.5 days-of-age for white-tailed deer) and were more closely aligned with survival estimates of known-age newborn fawns.

Neonatal-age models with positive intercepts and larger upper-range age estimates (i.e., 9.2–15.2 days-of-age for white-tailed deer and 19.8 days-of-age for mule deer) required new-hoof growth measurements of ≤0 mm for a neonate to be 0 or 1 day old. Estimates of 30-day survival using these positive intercept models differed from known-age survival estimates. Thus, intercepts may partially explain differences in survival estimates based on ages derived from models and those for known-age neonates. Similarly for mule deer, estimates of 30- and 120-day survival from the Robinette equation aligned with that for neonates using known ages; the negative intercept of the Robinette equation indicated an expected 6.3 mm of new-hoof growth for newborns (≤24-hrs old). The large positive intercept for mule deer in the Haskell equation required neonates to have a new-hoof growth measurement of –5.29 mm to be ≤24-hrs old, and resulted in an age discrepancy with known ages of 15.1 days and a depressed estimate of 30- and 120-day survival. Consequently, intercepts of equations to estimate age based on new-hoof growth play a key role in accurate estimation of age for neonatal ungulates. Additionally, differences in intercepts and growth rates (slopes) among neonatal-age models and variation in new-hoof growth among study sites and years in our study support the hypothesis that relationships between new-hoof growth and age may be population- and time-specific [17].

Variation in intercepts among growth models may be a function of sampling variance among biologists when measuring new-hoof growth. For example, based on data from the Brinkman equation, a 0.75-mm change (error) in measurement of new-hoof growth at birth would result in a 40.4% change in estimate of the regression intercept. This would result in an intercept similar to the Sams equation. Measurement error could be reduced within studies by having a single person conduct all new-hoof growth measurements, or at a minimum have a single person train all personnel. However, measurements across studies likely result in variance caused by observer bias that contribute to differences in intercepts. Also, variation in intercepts may possibly be explained by biological variance such as gestation length and physical condition of the mother [58]–[60]. Increasing birth weight is related to longer gestation period [58] while increasing body mass is correlated with greater hoof growth [19]. Gestation period can range dramatically for ungulate species [58]–[60] and may be shortened or lengthened depending upon nutritional status of the female and environmental conditions [14], [61]–[63]. Additionally, new-hoof growth of white-tailed deer differed between females fed a high- and low-protein diet; neonates born to females on a low-protein diet had shorter hoof-growth measurements than those born to females on a high-protein diet [19]. Other ancillary variables such as maternal nutritional condition, birth mass, and litter size also may contribute to differences in new-hoof growth and thus, intercepts in equations for estimating age.

Probability of survival can fluctuate markedly with age during the first weeks of life for neonatal ungulates and generally is thought to occur because of size, agility, activity, and vulnerability of neonates [5], [12]–[14], [64]. Our results indicate that discrepancies in estimates of age from the true age of wild-captured neonates can alter results of temporal survival patterns and thus, interpretation of factors influencing survival. For example, initial observations suggested the greatest period of vulnerability for white-tailed deer in Minnesota and South Dakota aged using the Brinkman equation occurred during the first 2 weeks-of-life [13]. These results were consistent with Rohm et al. [5] who aged fawns using the Haugen and Speake equation and attributed the greatest period of mortality to changes in habitat availability and coyote (*Canis latrans*) behavior. Conversely, the same neonates aged using the Haskell equation would not support these conclusions but rather supported Nelson and Woolf [11], who observed that neonate mortality was highest during 2–8 weeks-of-life. They [11] hypothesized that neonates were safe from predation because of their sedentary behavior when 0–2 weeks old, were most vulnerable when neonates became active during 2–8 weeks old, and could evade predators when >8 weeks old. Understanding the behavior of wild, young ungulates, their vulnerability to mortality, and assessing the influence of management actions on probability of survival could be undermined by using inaccurately aged neonates in survival analyses.

The advent of powerful modeling techniques available in Program MARK permits the use of individual covariates such as birth weight to examine their influence on survival [5], [13], [14], [42]. Weight at birth is a key factor affecting probability of survival for young ungulates because it is associated with strength and viability of neonates [10], [19], [64]–[66]. Estimates of birth weight calculated using weight at capture and estimated age to back-calculate that metric [5], [41], [67], would vary depending on the hoof-growth equation used. However, this should have little effect on model results using estimated birth weight as a covariate, unless overestimation of ages is large enough to yield estimates of birth weight that are truncated at 0 kg (i.e., Haskell for white-tailed and mule deer). Without this truncation, the relationship between survival and estimated birth weight will remain stable because larger neonates will have a greater probability of survival than smaller neonates even if absolute values for estimated birth weight are biased upward or downward for all individuals. Consequently, caution should be used if management objectives include identifying a threshold in birth weight below which fawn survival is compromised, because thresholds will be biased low when ages at capture are overestimated.

Timing of parturition coincides with the flush of nutrients during the spring to support the costs of lactation [10], [68], allow sufficient growth and accumulation of body reserves of young before winter [69], potentially avoid high predation pressure [12], [70], and enhance survival of young. Although other methods for determining peak parturition are available including evaluation of movement with GPS data or use of VITs [38], [39], [71], most studies rely on back-calculated birth dates from estimated age-at-capture to determine parturition dates [10]. As with estimated birth weights, relationships between parturition dates and other characteristics will remain relative, but identifying actual dates for peak parturition or thresholds in those mathematical relationships could be biased by inaccurate neonatal-age models. For example, Lomas and Bender [10] observed an 18–29 day shift in mean birth dates of mule deer in north-central New Mexico between the 1980s and the early 2000s in response to a marked decline in habitat quality in the region. Similar apparent shifts could be noted between studies that use different models to estimate ages of neonates even if little change had actually occurred.

## Conclusions

Survival of young drives annual population trajectories for ungulates and influences management strategies for harvest, habitat treatments, and predator control [67]. Survival of neonates is routinely estimated through capture and collaring during the first few weeks of life, with subsequent monitoring for survival. Neonatal-age models based on new-hoof growth have been regarded as the most accurate method to back-calculate date of birth, but as we demonstrated, choice of model can have a profound effect on age-dependent patterns of mortality and short-term estimates of survival. Our results indicated that estimates of summer survival were more robust to variation in estimates of age-at-capture and support the reliability of most previously reported estimates of survival that used neonatal-age models. We encourage researchers to use caution, however, when interpreting estimates of survival, birth weights, and parturition dates when age is estimated based on hoof-growth equations because some models perform better than others. In most studies, a portion of wild-captured neonates may be confidently identified as newborn either through observation of birth or robust criteria such as that used in our study. Therefore, we suggest testing for differences in birthdates and estimated birth weights between known-age neonates and those whose ages are estimated to assess the potential for bias associated with those hoof-growth equations [14]. Alternatively, researchers could estimate their own growth models [17] if sufficient data were available. Finally, our analyses indicate that modeling survival in daily intervals is too fine a temporal scale when birth date is unknown because of the potential inaccuracies among models available to estimate age of neonates. We suggest that weekly survival intervals are more appropriate because most hoof-growth models accurately predicted neonatal age within one week.
